# Addressing 6 challenges in generative AI for digital health: A scoping review

**DOI:** 10.1371/journal.pdig.0000503

**Published:** 2024-05-23

**Authors:** Tara Templin, Monika W. Perez, Sean Sylvia, Jeff Leek, Nasa Sinnott-Armstrong

**Affiliations:** 1 Department of Health Policy and Management, University of North Carolina at Chapel Hill, Chapel Hill, North Carolina, United States of America; 2 Carolina Population Center, University of North Carolina at Chapel Hill, Chapel Hill, North Carolina, United States of America; 3 Department of Genome Sciences, University of Washington, Seattle, Washington, United States of America; 4 Department of Health Policy and Management, University of North Carolina at Chapel Hill, Chapel Hill, North Carolina, United States of America; 5 Sheps Center for Health Services Research, University of North Carolina at Chapel Hill, Chapel Hill, North Carolina, United States of America; 6 Biostatistics Program, Fred Hutchinson Cancer Center, Seattle, Washington, United States of America; 7 Department of Biostatistics, University of Washington, Seattle, Washington, United States of America; 8 Herbold Computational Biology Program, Fred Hutchinson Cancer Center, Seattle, Washington, United States of America; Washington University in Saint Louis, UNITED STATES

## Abstract

Generative artificial intelligence (AI) can exhibit biases, compromise data privacy, misinterpret prompts that are adversarial attacks, and produce hallucinations. Despite the potential of generative AI for many applications in digital health, practitioners must understand these tools and their limitations. This scoping review pays particular attention to the challenges with generative AI technologies in medical settings and surveys potential solutions. Using PubMed, we identified a total of 120 articles published by March 2024, which reference and evaluate generative AI in medicine, from which we synthesized themes and suggestions for future work. After first discussing general background on generative AI, we focus on collecting and presenting 6 challenges key for digital health practitioners and specific measures that can be taken to mitigate these challenges. Overall, bias, privacy, hallucination, and regulatory compliance were frequently considered, while other concerns around generative AI, such as overreliance on text models, adversarial misprompting, and jailbreaking, are not commonly evaluated in the current literature.

## Introduction

Artificial intelligence (AI) systems have expanded in popularity in the past 2 years as hardware, training, and methodological improvements result in better-than-human performance on many tasks [1,2]. Generative AI tools that create text, images, and other content are already being deployed in many medical settings, and anyone with an internet connection is able to access ChatGPT. Simultaneously, many medical institutions are evaluating AI to assist with tasks that humans find tedious or time-consuming. Current research underscores the productivity enhancements brought about by these generative AI tools, especially among new employees [3–5].

Despite the potential of generative AI for many applications in healthcare, digital health practitioners must understand these tools and their limitations [6–8]. Generative AI can exhibit biases [9,10], compromise data privacy [11,12], misinterpret prompts[13,14], and produce hallucinations [15]. Given the rapid uptake and integration of this technology, failure to appreciate their current limitations can lead to misuse and, ultimately, patient harm and other unintended consequences [6]. We conducted a scoping review on the state of generative AI for medicine in March 2024, with the goal of identifying important areas of discussion in the literature. We chose this review format as it captures the overall trends in this rapid emerging area with limited primary literature available. We specifically identified 6 challenges with generative AI and sought to evaluate when, how, and why these were presented in the medical literature, with the goal of unifying these multiple components and clarifying where the field might need to place additional effort.

**Fig 1:**
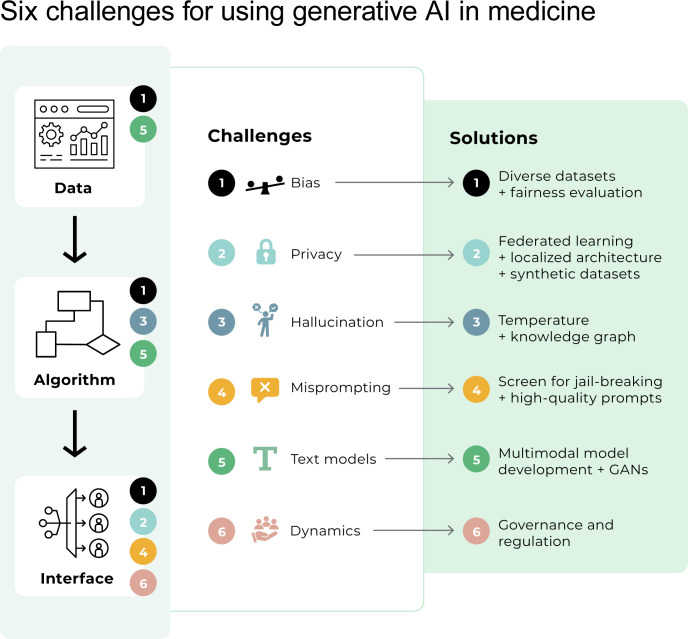
Six challenges for using generative AI in digital health. Despite the potential of generative AI for many applications in healthcare, experts must understand these tools and their limitations. Here, we present an abstraction of an AI system (Training Data, Algorithm, and Interface) and key challenges with each part of the system. All parts of the system must be evaluated for bias (Challenge 1). Most training data and model development have focused on text (Challenge 5), potentially missing opportunities for multimodal model development and generative adversarial networks. The generative AI algorithm may hallucinate or produce inaccurate or nonsensical output (Challenge 4). Finally, issues impacting interfacing with generative AI technologies include maintaining privacy (Challenge 2), protecting the model from adversarial attacks (Challenge 4), and regulating dynamic behavior (Challenge 6). GAN, Generative Adversarial Network.

**Fig 2:**
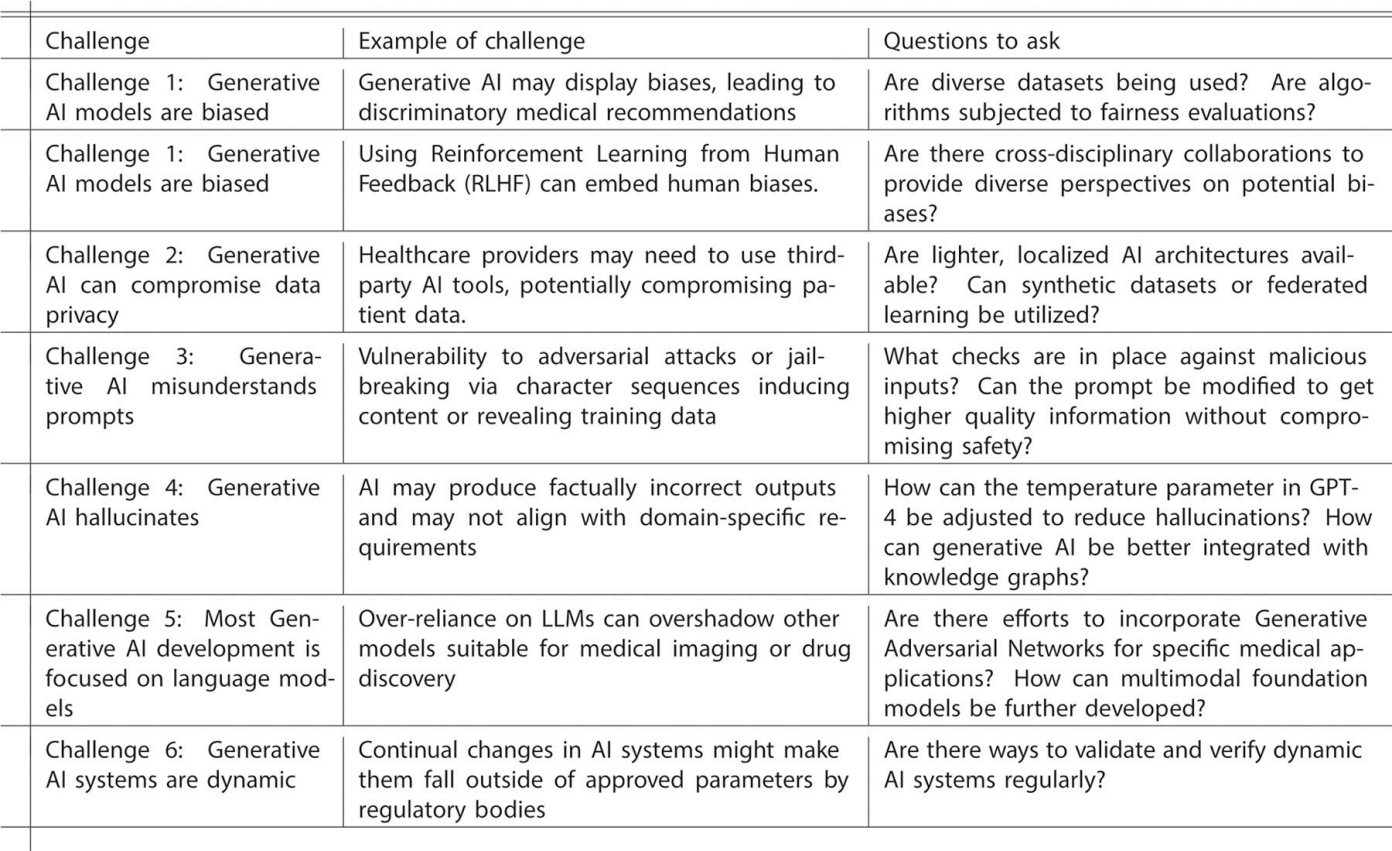
Examples of challenges and key questions to ask. This table presents examples of each challenge and questions to ask.

After first discussing general background on generative AI, we focus on collecting and presenting these 6 challenges key for digital health practitioners and specific measures that can be taken to mitigate these challenges. We summarize these challenges and some potential solutions in Fig 1 and provide examples in Fig 2.

## Background

Generative AI encompasses AI techniques designed to create new content [16]. This content can range from images, videos, and text to more specialized outputs such as 3D models, genomic sequences, or medical diagnostics. The basic idea of generative AI is to model the underlying data distribution so that new instances can be generated that are statistically similar to the original data [17]. A notable subset of generative AI technologies is large language models (LLM). LLMs refer to a type of artificial intelligence model that has been trained on text data—such as books, articles, and websites—to generate novel text.

Commonly used generative AI models have extensive tools developed around them, making it easier for digital health practitioners to incorporate them into projects. GPT-4 (Generative Pre-trained Transformer 4) was developed by OpenAI [18]. It’s mainly used for text generation but can also be fine-tuned for various tasks (for example, translation) and now incorporates image generation. ChatGPT is a version of the GPT series fine-tuned specifically for conversation and available via an online chat dialog interface. There are also models that make use of more specialized data. One example is Med-PaLM from Google, which is trained on medical data [19]. LLaMA (Language Model for Many Applications, an open-source LLM from Meta) offers a resource-efficient alternative to GPT-4, compatible with less powerful hardware. Because it is open source, analysts can control everything if they wish to fine-tune the algorithm for specific data types. Being open source also decreases the risk of adversarial attacks against the model by enabling end-to-end verification.

Generative AI has the potential to change many aspects of digital health. While the implementation of systems has rapidly progressed, a number of ethical and legal challenges remain to the widespread, safe, and effective use of these tools. We performed a scoping review of challenges that may impact AI systems with proposed solutions for digital health practitioners. We believe understanding the perceptions of challenges in the field and collecting solutions from digital health practitioners and interdisciplinary collaborators will enable these technologies to thrive.

## Methods

We searched the PubMed database for articles that specifically discussed generative AI technologies and common challenges that have been discussed previously in review articles of challenges with AI technologies [20–22]. In early March 2024, we searched for all papers that contained the following string in either the title or the abstract: ("GPT" OR "llama" OR "transformers" OR "Generative AI" OR "Large Language Model" OR "ChatGPT" OR "Generative Adversarial Networks" OR "Variational Autoencoder" OR "Multimodal model") AND ("Bias" OR "Patient Privacy" OR "HIPAA" OR "Hallucination" OR "Prompting" OR "Jailbreaking" OR "Governance" OR "Oversight" OR "FDA" OR "GDPR") AND "Medicine". The general search strategy requires that the article discuss generative AI technology, one of the common concerns with these technologies, and medicine. We only reviewed articles in English and did not restrict the time frame or article type. We excluded articles that were about medical education or scientific writing, as they fall outside the scope of the study. Results without abstracts were excluded. Each paper was reviewed by 2 independent reviewers using a standardized data collection form available in S1 Appendix.

We extracted the following characteristics of the included articles: PubMed ID; challenges discussed; the article type (primary literature, model validation, opinion, review); the specific generative AI technology discussed (ChatGPT; GPT-3; GPT-3.5; GPT-4; OpenAI; MedPalm; Llama 2; Bard/Gemini; LLMs (in general); generative AI (in general); generative adversarial networks; variational autoencoder; multimodal models); if there was a specific use case or subfield of medicine; and we recorded if the paper suggests common recommendations for AI best practices. Differences in reviewer responses were resolved by taking the union of their responses. The scoping review plan was not preregistered in this study, and we provide other reporting items in S1 PRISMA Checklist.

Because this is not human subjects research and was a review of previously published articles, the study did not require the approval of an Institutional Review Board.

## Results

Our initial search yielded 173 unique papers (S1 Fig). We excluded 53 papers due to missing abstracts or because the focus was off our topic of challenges in generative AI for the practice of medicine. This process resulted in 120 papers being included in this review. We found that 52% (*N* = 62) were primary literature (e.g., a data collection effort, such as an audit), 28% (*N* = 34) were review articles; 23% (*N* = 28) were opinion pieces, and 12.5% (*N* = 15) were model validation papers presenting a new generative AI model (S2 Fig). We classified 17% of papers (*N* = 20) into multiple categories. Although we did not restrict the article publication date, we found that the earliest paper was published in April 2021, and the majority of articles were published after September 2023 (S3 Fig). We found the majority of articles addressed model hallucinations (*N* = 77, 64%) and bias (*N* = 69, 58%), followed by privacy (*N* = 39, 33%), regulation (*N* = 37, 31%), misprompting (*N* = 9, 7.5%), and, finally, overreliance on text models (*N* = 8, 6.7%) (S4 Fig). Bias and hallucination were the most likely to be mentioned together in an article (*N* = 16), followed by bias and privacy (*N* = 8), regulation and hallucinations (*N* = 8), and all 4 topics together (*N* = 8) (S5 Fig).

### Challenge 1: Generative AI models are biased (interface/algorithm/data)

Understanding bias in machine learning is critical, particularly given the history of machine learning models trained on biased data that lead to discriminatory and flawed medical recommendations [10,23]. Early versions of LLMs such as GPT-2 displayed similar biases [24]. During our review of papers, we identified 69 papers (57.5%) that raised bias as a concern for medical practitioners or patients using AI assistance for medical decisions.

To counteract this, model providers employed debiasing techniques by preprocessing the training data to remove bigoted content, altering the algorithm itself to incorporate human feedback, and postprocessing the model’s predictions. The efficacy of these techniques is still a subject of debate [25–28]. For instance, certain debiasing methods might correct for one form of bias but introduce another, largely because bias measurement and evaluation can vary across methods. As an example, a proportional representation metric may indicate that one group of individuals is underrepresented in training data. A reweighting scheme might be used to mitigate bias due to this underrepresentation, but reweighting may then degrade model performance for other groups, such as what happened with Gemini’s widely publicized image generation [29]. Moreover, debiasing techniques may not account for more complex, intersectional forms of bias that involve multiple attributes like race, gender, and age.

### Opportunities

For digital health practitioners concerned about bias in AI, actionable steps include subjecting algorithms to rigorous, multidimensional fairness evaluations and considering guidelines put out by groups such as FaaCT (facctconference.org) and Coalition for Health AI (coalitionforhealthai.org). In our review, we found a theme of researchers across a broad range of medical subfields evaluating ChatGPT’s responses for the accuracy of its medical advice across patient attributes, using varied audit methods and accuracy metrics [30,31].

Many papers that we reviewed (*N* = 49, 41%) called for cross-disciplinary collaboration with ethicists, social scientists, and domain experts to provide important perspectives on potential bias [32–35]. Incorporating human feedback, through reinforcement learning from expert feedback (RLEF) and reinforcement learning from human feedback (RLHF) can also be used to mitigate some of these concerns [36], though the potential for human biases in this process should be carefully considered. Implementing regular audits of AI models focused on ethical AI, such as one recently performed in *PLOS Digital Health* [37], could also help keep the technology in check [38]. Many papers (*N* = 42, 35%) also called for transparency in methodology and open sharing of debiasing techniques and evaluation metrics to foster collective progress in building more equitable AI systems.

### Challenge 2: Generative AI can compromise data privacy (interface)

Generative AI models often contain billions of parameters that require significant computational power to generate accurate responses. As a result, resource-limited labs or healthcare providers may be compelled to rely on external, third-party digital tools for computational support. However, there are ethical, regulatory, and patient privacy concerns with using third-party generative AI tools. Before sensitive data are uploaded into these tools, potential users must conduct a thorough legal and data privacy review, which itself is resource-intensive.

### Opportunities

Institutions face crucial trade-offs about the infrastructure they employ when it comes to privacy. On one hand, third-party "Software-As-A-Service" tools are easy to deploy, capable of handling large models, and include continual updates. Additionally, these services are externally managed, alleviating pressure to set up and maintain infrastructure. However, there may be fewer privacy concerns if institutions pursue local hosting of AI models due to significantly more control over the data usage and compliance with law. However, this requires dedicated infrastructure, security measures, and knowledgeable local personnel.

Only 7 papers (5.9%) suggested localized architecture as a specific opportunity to mitigate privacy concerns in the context of generative AI. However, developers are creating "lighter" architectures that have fewer than 10 million parameters, can run on local networks or mobile devices, are optimized for specific tasks, and can be trained in less time than larger models, using a combination of model compression and higher-quality training data [39–41]. Using generative AI models locally lessens privacy risks, as the data never leave the secure local network or device [13], though there are still many other concerns [11]. Hardware specialized for these types of models (from graphical processing units to Internet of Things wearables) is also being developed to optimize local model runtime and battery life [42]. While the adoption of wearables for healthcare has been low [43], patients and physicians may increase adoption as wearables offer more value in improved digital health and telemedicine.

In response to patient privacy concerns and acknowledgment that models may not perform well in unique patient populations [44], there is interest in federated learning [45,46]. Federated learning is when multiple actors (for example, multiple independent hospital systems or multiple Internet of Things devices) collaboratively train a model by exchanging model updates without sharing patient data. This approach maintains data privacy and keeps patient data local but enables clinicians to benefit from models trained on more patient records. There were no studies in our review that mentioned federated learning as a key opportunity. Further work is needed to develop federated learning methods for generative AI technologies in clinical practice [47].

### Challenge 3: Generative AI misunderstands prompts (interface)

Due to ChatGPT’s popularity and ease of use, the chatbot interface and the importance of crafting effective prompts have reached mainstream attention (although not all generative AI models are text-based). There remain gaps in knowledge on how to effectively prompt these technologies, at both basic and advanced levels. For example, although patients may be able to query ChatGPT about their health (thus democratizing access), they may be substituting that for necessary medical advice [48,49]. Most current resources are commercial guides focused on specific products, which may not address the unique requirements of prompting for medical practice.

Finally, both open-source and closed-source LLMs are vulnerable to specific character sequences that can induce harmful, biased, or unintended content in response to user prompts (called adversarial attacks or jailbreaking). For example, some adversarial attacks can recover training data (such as personally identifiable information [12]). It is uncertain if such behavior can be mitigated by LLM providers [13].

### Opportunities

Thirty-nine papers (33%) highlighted the importance of practitioners understanding some heuristics for crafting effective prompts [50,51]. The specifics of prompting will continue to evolve over the long term as we learn more about these models [52,53]. Eventually, LLMs may become better at articulating what the user wants than the user [54].

Of the *N* = 120 papers that we reviewed for this analysis, only 3 mentioned jailbreaking as a concern for generative AI technologies in medical settings. Jailbreaking has long been a concern in fields such as cybersecurity, and practitioners of digital health need to be aware of this threat. LLMs can be used to jailbreak other LLMs, and often due to the large size, retraining models to patch vulnerabilities is nonfeasible [55]. Algorithms to reduce adversarial attacks and ensure the responses align with human values should be deployed in medical settings [56,57].

### Challenge 4: Generative AI hallucinates (algorithm)

Many types of AI models generate outputs—or hallucinations—that are factually incorrect. This may occur when the model emphasizes certain parts of the input while neglecting other (potentially more relevant) parts or if there are errors in the training data. LLMs are fundamentally a series of mathematical transformations based on statistical patterns, not a conscious process. Professionals embedded in clinical processes may lack foundational AI training to adequately address hallucinations [58]. Conversely, those adept in AI often miss the nuanced domain-specific knowledge crucial for crafting AI-assisted diagnostic tools. This disconnect and lack of multidisciplinary expert review pose risks in creating systems that might misinterpret or inaccurately represent biomedical data due to these hallucinations.

### Opportunities

Similar to papers evaluating model bias, there was a theme among papers evaluating model hallucinations of researchers evaluating ChatGPT’s responses for accuracy across medical subfields [59]. The consensus of these papers was that physicians should review medical advice to patients and not rely on an AI for assistance. Similar to bias, the most recommended solution to hallucinations was an external review by experts (*N* = 49, 41%). However, there were some tasks where hallucination was less of a risk, and, thus, physician oversight was not needed: lowering the reading level of already approved medical advice for patients, finding and extracting social determinants of health in the medical record, etc.

Only *N* = 11 (9.2%) of papers suggested modifying model parameters to address hallucination. Practitioners should be aware of the temperature parameter in GPT-4, which adjusts the model’s output randomness; higher temperatures result in more “creative” responses (with more potential for hallucination), whereas lower temperatures yield focused results closer to the training data and prompts [60]. Adversarial testing or out-of-distribution evaluation can be helpful in mitigating such hallucinations for developers. In applications such as AI-assisted diagnosis, introducing an expert-in-the-loop can help in identifying and correcting hallucinations.

There is demand for an AI that accurately searches a knowledge graph (e.g., academic abstract databases like PubMed) and produces citations or linked references [61–63]. We further anticipate that integration with existing knowledge will become common in generative AI systems.

### Challenge 5: Most generative AI development is focused on language models (algorithm/data)

Medical practice incorporates a wide range of data types, including imaging, genetic sequences, biometric data, and more. Of the 120 papers that we reviewed, 81% (97 articles) were about ChatGPT, GPT-4, or other LLMs produced by OpenAI (S6 Fig). However, other generative models could be better for medical applications such as medical imaging or drug discovery. By focusing mainly on LLMs, we might miss opportunities presented by these alternative models.

### Opportunities

There are many emerging uses of generative AI with nontext data. In genetics and pharmaceutical research, generative AI can analyze the chemical structures of existing drugs (using, e.g., SMILES [64], and generate new molecular structures that are likely to have desired therapeutic effects. Generative adversarial networks can also generate synthetic data, helpful for protecting patient privacy and harnessing the generative capabilities [65]. Despite the focus on OpenAI’s models, our review also uncovered other models in development that may be of interest to digital health practitioners [66,67].

There is particular interest in multimodal foundations models—AIs that can interpret and generate multiple types of data simultaneously—which may enhance clinical practice [68]. Clinicians often dictate clinical notes during or after patient visits. Generative AI could take these voice recordings and annotate them for specific medical terms or highlight potential areas of concern. The annotations could then be used for coding diseases, generating billing information, flagging potential conditions for further investigation, or quality assurance. It is also possible to generate text output from nontext input, which may aid in generating alt text representations of images for accessibility [69,70].

### Challenge 6: Generative AI systems are dynamic (interface)

Generative AI agents are being used in clinical practice for a wide variety of tasks [71,72]. Systems of AI agents working together may become common in the workplace [54]. These agents do not follow hard-coded rules but rather adapt and make decisions based on their "experiences" and "interactions" within the system [73]. This allows for more dynamic, emergent behaviors and outcomes, which can provide deeper insights into complex systems, such as oncology trials [74]. However, medical devices, including AI, need to be approved by regulatory bodies, which requires proving that they are safe and effective [75], yet many have not been approved. If an AI system is continually changing, it might not remain within the approved parameters. Ongoing evaluation is critical in applications where data drift may be a concern [76–78].

### Opportunities

Much has been written about the urgency of regulation. In short, there are issues of compliance with the Health Insurance Portability and Accountability Act (HIPAA) and General Data Protection Regulation (GDPR), as well as issues of responsibility in medical malpractice [79,80]. Calling for regulation was a common recommendation of the literature we surveyed; 32% (*N* = 38) called for increased attention to HIPAA and GDPR compliance, and 35% (*N* = 42) called for greater transparency in the data inputs and outputs for regulation. While many articles called for greater regulation, there is still much debate about how to regulate this technology effectively [81]. We direct the interested reader to Bertalan Meskó and Eric Topol’s review [79], which was identified during our search and details a plan to regulate companies rather than specific models. We also direct the interested reader to the World Health Organization policy brief [82] and the United States government’s directive to the Food and Drug Administration to regulate AI in medical settings [83–85].

A few articles that we reviewed (*n* = 16, 13%) called for not using private health information to train models and, instead, using synthetic data sets (generated data that emulate real-world data) [86–89]. These data sets are gaining popularity in biomedical settings because they can facilitate research, including generative AI model training, in contexts where data are scarce or sensitive. These also naturally fit into deep learning approaches, like generative adversarial networks, which are designed to synthesize new data sets. As they become more common, practitioners need to know these data sets may be limited by the distributions observed in the original data (for example, limitations due to small training samples). An open area of development is how to replicate causal effects identified in the medical literature in synthetic data.

## Conclusions

Generative AI can exhibit biases, compromise data privacy, misinterpret prompts, and produce hallucinations. In this article, we performed a scoping review of challenges that may impact AI systems with proposed solutions for digital health practitioners. This review focused on generative AI approaches in 2022 through early 2024 and focused specifically on application areas that are already part of medical practice, as well as a subset of 6 core challenges that emerged as repeated themes in the literature. We have attempted to convince domain experts in digital health that, although there are challenges, with a grasp of these technologies, there are also opportunities. Seeking out diverse data sets and robust fairness evaluations can mitigate biases. Localized, domain-specific AI models bolster data privacy, while innovations in hardware and wearables may eventually enhance telemedicine. Adversarial testing, expert-in-the-loop mechanisms, and knowledge integrations can enhance prompting and limit hallucinations, eventually enhancing the clinical processes. While much AI focuses on language models, huge potential lies in nontext data applications. Lastly, renewed attention on regulation will both clarify appropriate use within clinical practice and encourage innovations around synthetic data that are HIPAA compliant. Digital health technologies will likely improve by understanding the perceptions of challenges in the field and collecting solutions from digital health practitioners and interdisciplinary collaborators.

## Supporting information

S1 Appendix The structured data collection form used for recording information about each paper evaluated in the scoping review, including the PMID, challenges addressed, type, technologies used, field, and recommendations. (DOCX)

S1 PRISMA Checklist The Preferred Reporting Items for Systematic reviews and Meta-Analyses extension for Scoping Reviews (PRISMA-ScR) checklist, indicating individual document sections for reporting of each checklist item. (DOCX)

S1 Fig PRISMA statement flow diagram. (EPS)

S2 Fig Classification of each paper found in the scoping review into primary literature, review articles, opinion pieces, and model validation. (EPS)

S3 Fig Time of publication of each paper found in the scoping review. (EPS)

S4 Fig Classification of each paper found in the scoping review into the 6 challenges: bias, privacy, hallucination, misprompting/jailbreaking, text models, and dynamics/regulation. (EPS)

S5 Fig Overlap of challenges mentioned in each paper found in the scoping review. (EPS)

S6 Fig Classification of each paper by type of generative AI technology evaluated. (EPS)
