# Supplementary material for: Addressing 6 challenges in generative AI for digital health: A scoping review
Source: PLOS Digit Health. 2024 May 23;3(5):e0000503. doi: 10.1371/journal.pdig.0000503 (PMC11115971; doi:10.1371/journal.pdig.0000503)
Supplement: S1 Appendix — (DOCX) [file pdig.0000503.s001.docx]

**Appendix**

**Structured Data Collection Form**

**Reviewer name:**

**PMID:**

**ALL PAPERS: Which challenge(s) does this paper address?**

**__ Bias**

**__ Privacy**

**__ Hallucinations**

**__ Misprompting/Jailbreaking**

**__ Text models vs Non-text**

**__ Regulation / Dynamics**

**__ EXCLUDE**

**ALL PAPERS: Is this primary literature, model validation, opinion, review?**

**__ Primary literature (e.g. an audit, data collection)**

**__ Model validation (e.g. new model vs SOTA)**

**__ Opinion**

**__ Review**

**ALL PAPERS: Which specific generative AI technology does this paper evaluate or discuss?**

**__ ChatGPT; GPT-3; GPT-3.5; GPT-4; OpenAI**

**__ MedPalm**

**__ Llama 2**

**__ Bard/Gemini**

**__ Large Language Models (in general)**

**__ Generative AI (in general)**

**__ Generative adversarial networks**

**__ Variational Autoencoder**

**__ Multimodal models**

**__ Other: [state if other]**

**ALL PAPERS: Does the paper address a specific use case or subfield of medicine? (we need to generate specific example of our challenges):_____**

**ALL PAPERS: Tally up proposed recommendations from experts:**

**__ [BIAS] Representative and diverse datasets**

**__ [BIAS/PRIVACY] Pre-process the input datasets (e.g. to remove some content - like PHI or hate speech)**

**__ [BIAS/HALLUCINATION] Adjust parameters of the genAI model (e.g. temperature)**

**__ [BIAS/REGULATION] Transparency in methods/tracking all inputs or outputs**

**__ [BIAS] Computational/quantitative fairness evaluation of the output (internal audit)**

**__ [BIAS] External audit with experts/Reinforcement Learning Expert Feedback/RLHF**

**__ [PRIVACY] Federated learning**

**__ [PRIVACY] Localized architecture**

**__ [PRIVACY/REGULATION] Synthetic patient data/Not using PHI to train**

**__ [PRIVACY] Encryption**

**__ [MISPROMPTING] Screening for malicious inputs/jailbreaking**

**__ [MISPROMPTING] Training people how to prompt GenAI for better results**

**__ [PRIVACY/REGULATION] HIPAA / GDPR compliance**

**__ Other: [state]**
